# Supplementary material for: Future changes in coastal upwelling and biological production in eastern boundary upwelling systems
Source: Nat Commun. 2024 Jul 24;15:6238. doi: 10.1038/s41467-024-50570-z (PMC11266572; doi:10.1038/s41467-024-50570-z)
Supplement: Supplementary file 1 — Supplementary Information [file 41467_2024_50570_MOESM1_ESM.pdf]

Supplementary Information for

Future changes in coastal upwelling and biological production in eastern boundary upwelling systems

Authors: Tianshi Du[1], Shengpeng Wang[1], Zhao Jing[1,2], Lixin Wu[1,2], Chao Zhang[3], Bihan Zhang[4]

Affiliations:

[1]Laoshan Laboratory, Qingdao, China

[2]Frontier Science Center for Deep Ocean Multispheres and Earth System (FDOMES) and Key Laboratory of Physical Oceanography, Ocean University of China, Qingdao, China

[3]FDOMES and Key Laboratory of Marine Environment and Ecology, Ministry of Education, Ocean University of China, Qingdao, China

[4]College of Marine Life Sciences, Department of Marine Ecology, Ocean University of China, Qingdao, China

Corresponding author: Shengpeng Wang (spwang1@qnlm.ac)

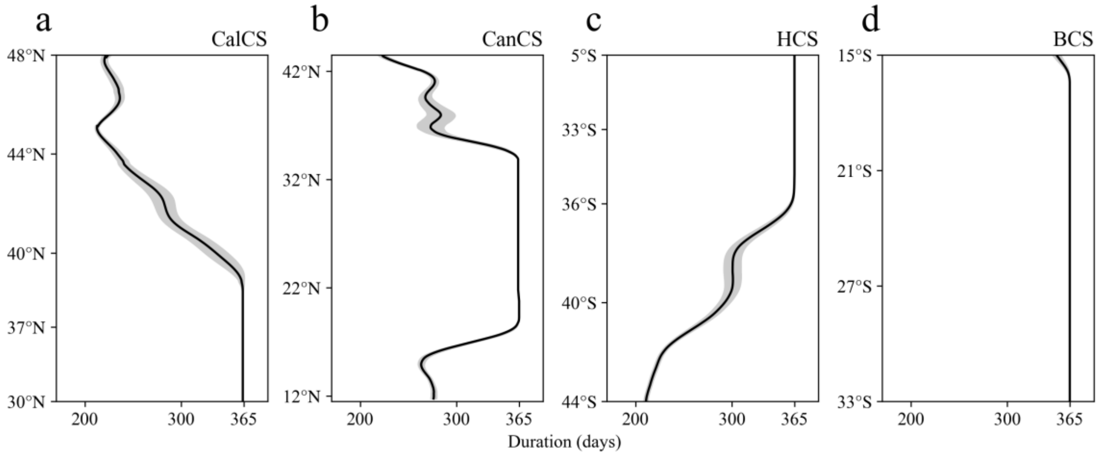

Supplementary Fig. 1. Latitudinal distribution of upwelling duration during 1920-1949. Upwelling duration in the California (CalCS, **a**), Canary (CanCS, **b**), Humboldt (HCS, **c**), and Benguela (BCS, **d**) Current Systems. The shading demonstrates the standard error across the 30-year coverage.

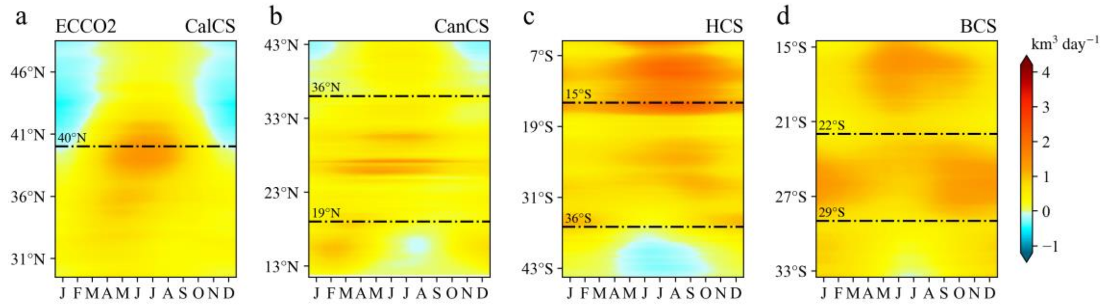

Supplementary Fig. 2. Upwelling index (UI) derived from ocean reanalysis dataset. Hovmöller diagram of the UI in California (CalCS, **a**), Canary (CanCS, **b**), Humboldt (HCS, **c**), and Benguela (BCS, **d**) Current Systems during 1992-2022 derived from Estimating the Circulation and Climate of the Ocean, Phase II (ECCO2).

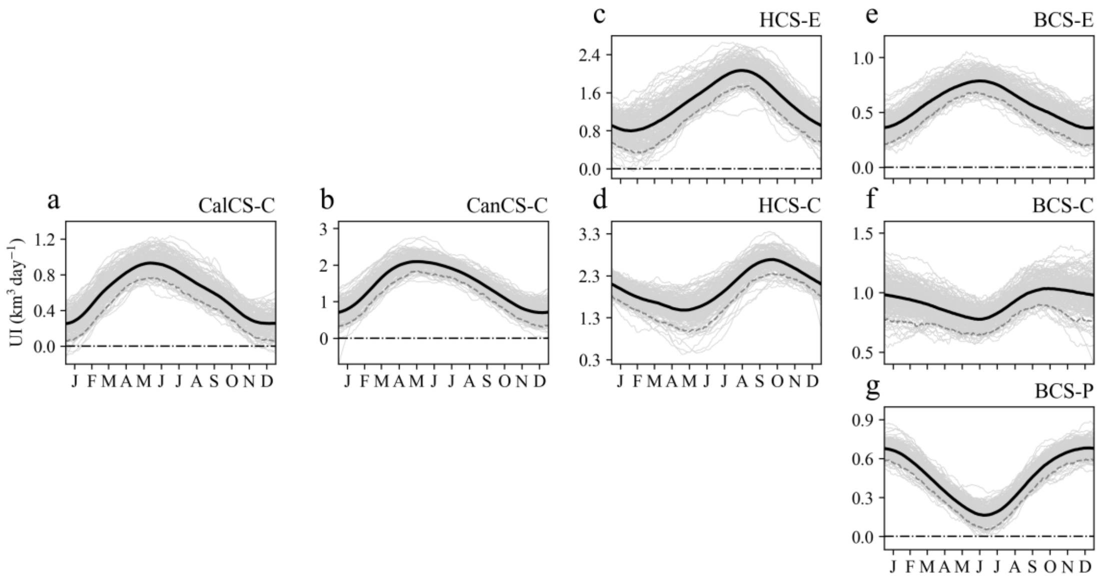

Supplementary Fig. 3. Upwelling index (UI) of upwelling regions with annual upwelling pattern. a, The averaged UI during 1920-2100 within the central California Current System (CalCS-C). The thin gray line denotes the UI for each year, while the black dotted line represents the 5th percentile of each year's UI. **b-g,** same as **a** but for results in the CanCS-C, HCS-E, HCS-C, BCS-E, BCS-C, and BCS-P. The 5th percentile line in each upwelling region exceeds 0, demonstrating persistent annual upwelling for more than 95% of the period.

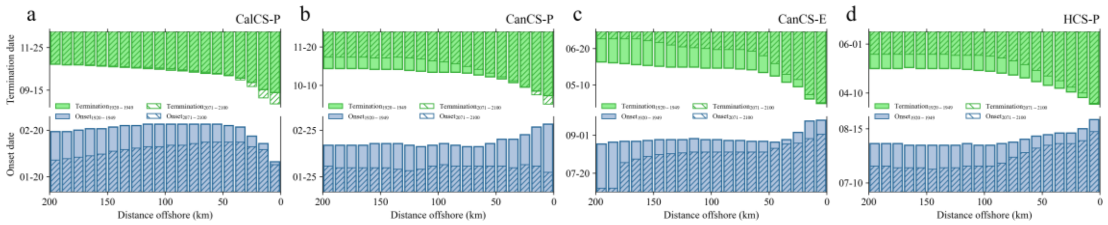

Supplementary Fig. 4. The climatology (colored bar) and future (hatched bar) upwelling onset and termination date in CalCS-P (**a**), CanCS-P (**b**), CanCS-E (**c**) and HCS-P (**d**). The x-axis denotes the average distance from the shore.

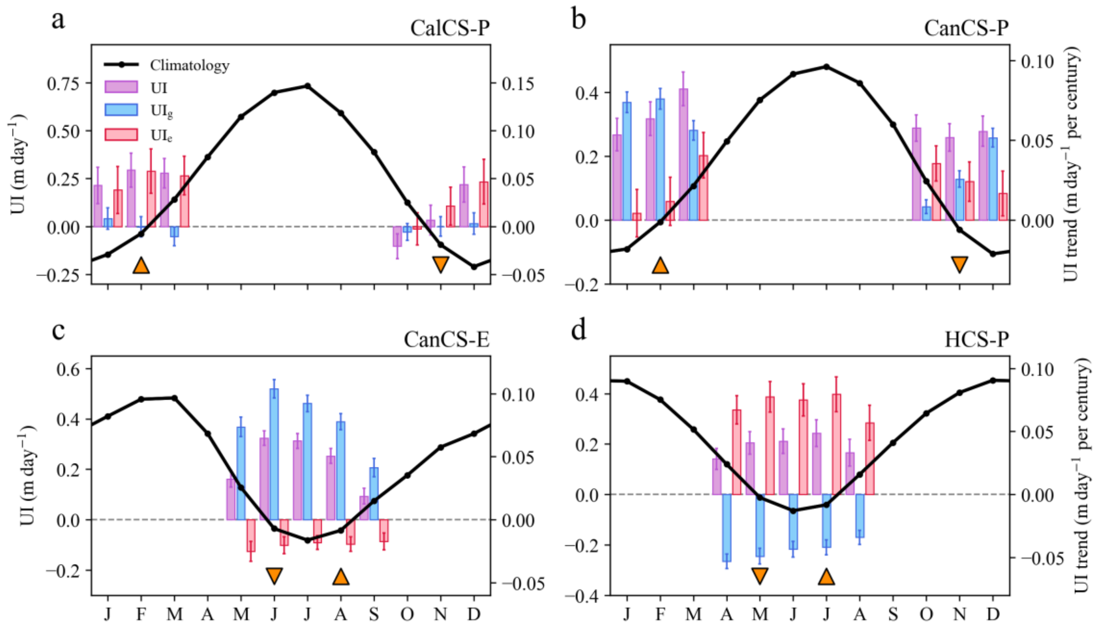

Supplementary Fig. 5. Climatology upwelling index (UI) and secular trends. a, the results derived from high-resolution Community Earth System Model simulation (CESM-H) in the poleward region of the California Current System (CalCS-P). The climatology UI (black) and UI trend during 1920-2100 (purple) near transition months. The wind-induced UI (UI_e) trend and geostrophic-induced UI (UI_g) trend were shown in red and blue. The orange triangle demonstrates the onset of upwelling while the inverted triangle represents the termination date. **b-d,** Same as **a** but for results in CanCS-P, CanCS-E, and HCS-P.

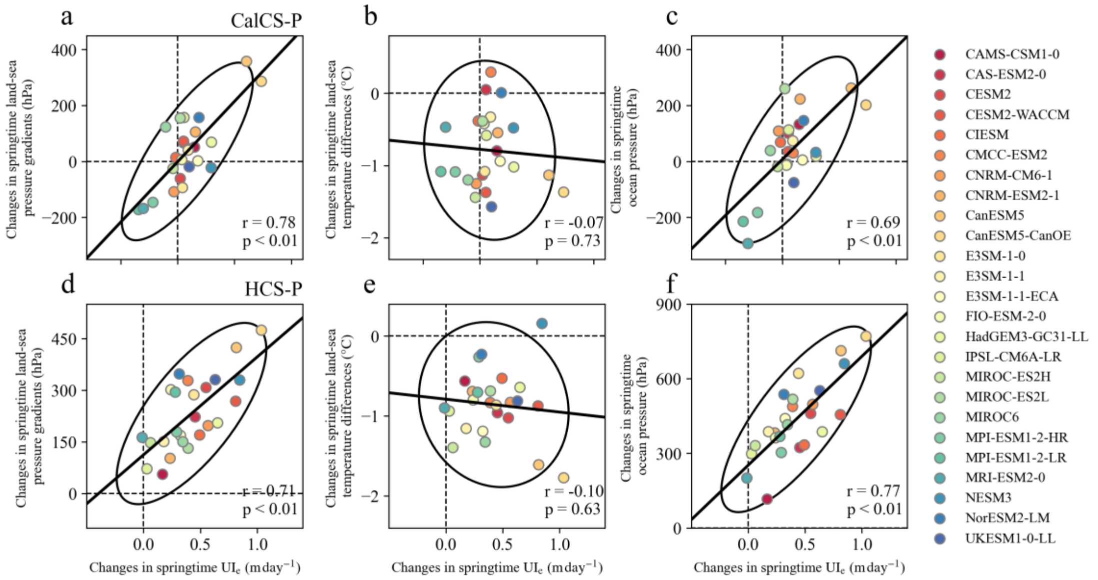

Supplementary Fig. 6. Causes of the springtime wind-induced upwelling index (UI_e) changes. The inter-model relationship between springtime UI_e and land-sea pressure gradients (**a**), land-sea temperature differences (**b**), and atmospheric pressure in the interior ocean (**c**) in the poleward California Current System (CalCS-P). Models were shown in symbols. The atmospheric pressure and air temperature in the interior ocean were averaged from 600 km offshore to approximately 2500 km offshore. Inland temperature and pressure were collected from the coastline up to 600 km inland. To assess the land-sea temperature differences and pressure gradients, inland values were subtracted from corresponding oceanic values [1]. Linear fit (solid black line) and 95% confidence ellipse are shown together with a correlation coefficient *r* and corresponding *P* value. **d-f,** Same as **a-c** but for results in the poleward Humboldt Current System (HCS-P).

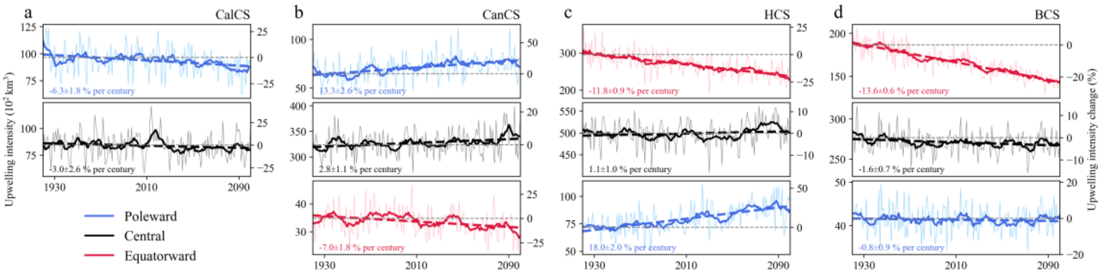

Supplementary Fig. 7. Same as **Fig. 3 a-d**, but for results averaged within 50 km offshore.

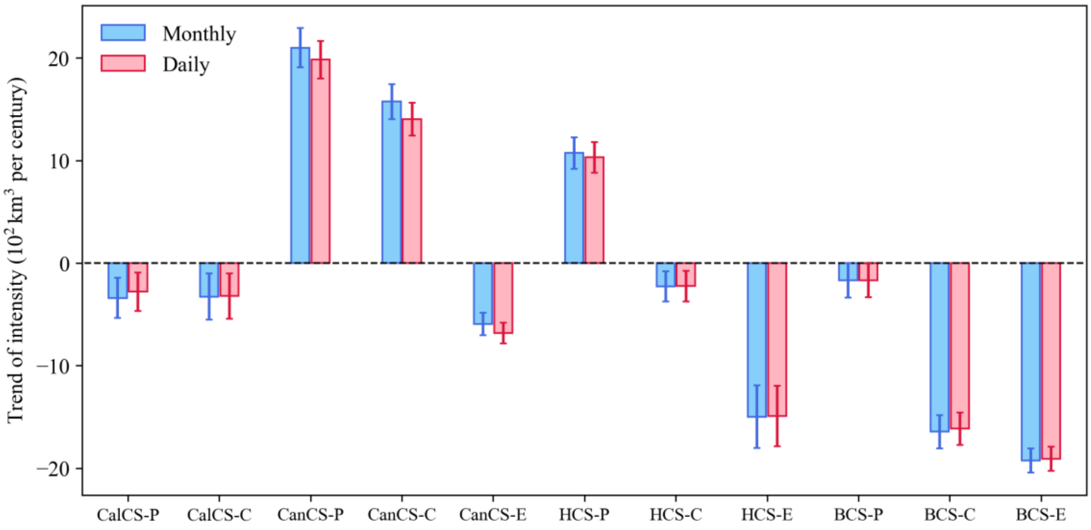

Supplementary Fig. 8. Influences of temporal resolution on evaluating secular changes in upwelling intensity. The upwelling intensity in each upwelling region is calculated via monthly (blue) and daily (red) outputs from the high-resolution Community Earth System Model simulation (CESM-H). The error bars denote the standard errors of the calculated slopes.

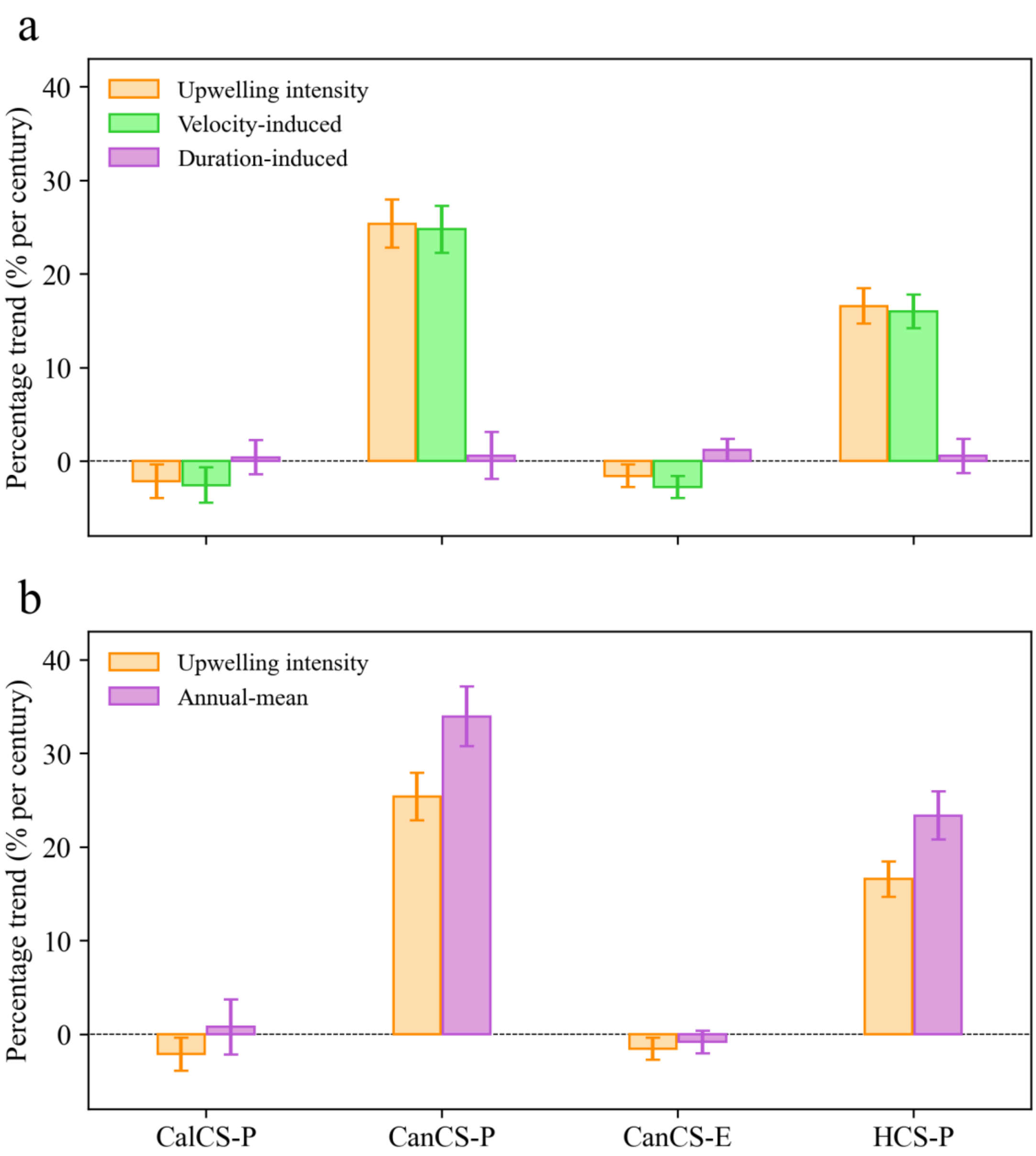

Supplementary Fig. 9. a, Trend of changing percentage in upwelling intensity (yellow), velocity-induced intensity (green), and duration-induced intensity (purple) in central California (CalCS-C), poleward Canary (CanCS-P), equatorward Canary (CanCS-E), and poleward Humboldt (HCS-P) regions during 1920-2100. **b,** Trend of changing percentage in upwelling intensity (yellow) and annual-mean upwelling (purple) during 1920-2100. The error bars denote the standard errors of the calculated slopes.

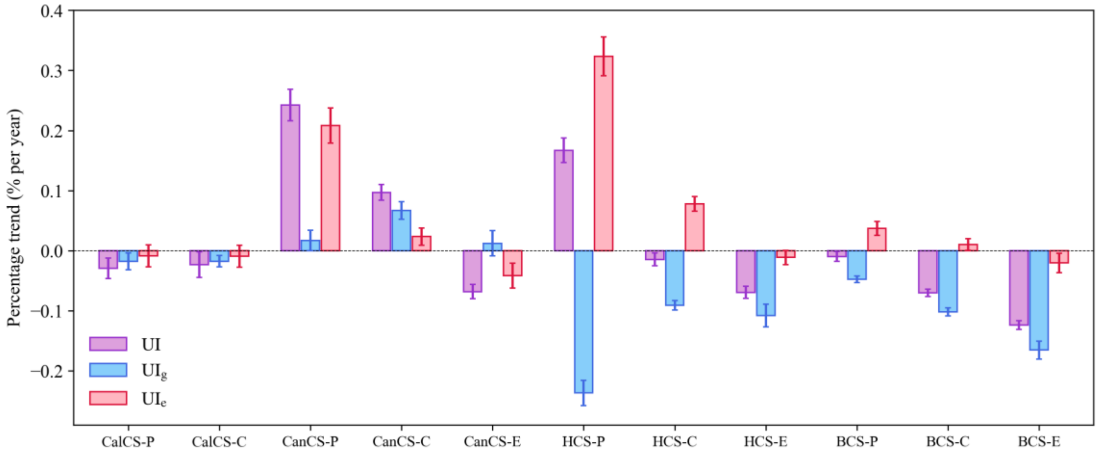

Supplementary Fig. 10. Decomposition of the long-term trend of upwelling intensity derived by high-resolution Community Earth System Model simulation (CESM-H). The upwelling intensity percentage trends calculated by upwelling index (UI, purple) decomposed to geostrophic-induced (UI_g, blue), and wind-induced (UI_e, red) within distinct upwelling regions.

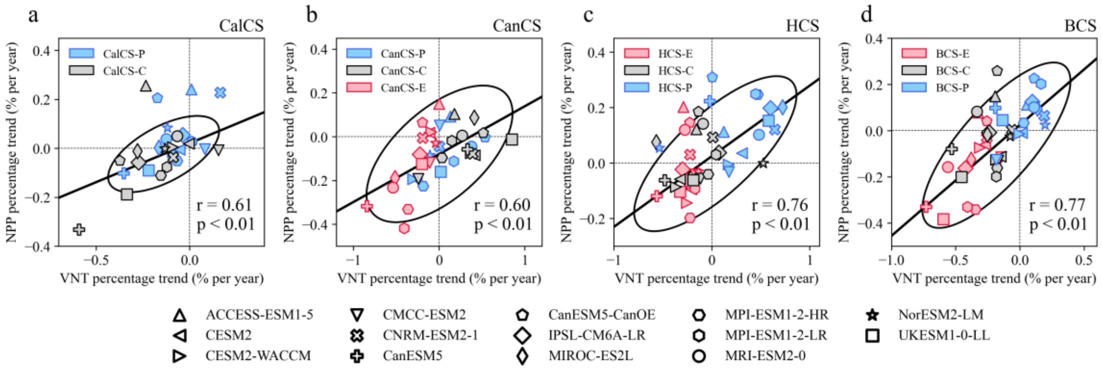

Supplementary Fig. 11. Relationship between vertical nutrient transport (VNT) and net primary production (NPP) within distinct eastern boundary upwelling system (EBUS). Same as **Fig. 4b**, but showing the California (CalCS, **a**), Canary (CanCS, **b**), Humboldt (HCS, **c**), and Benguela (BCS, **d**) Current Systems respectively. The equatorward, central, and poleward regions were colored red, gray, and blue.

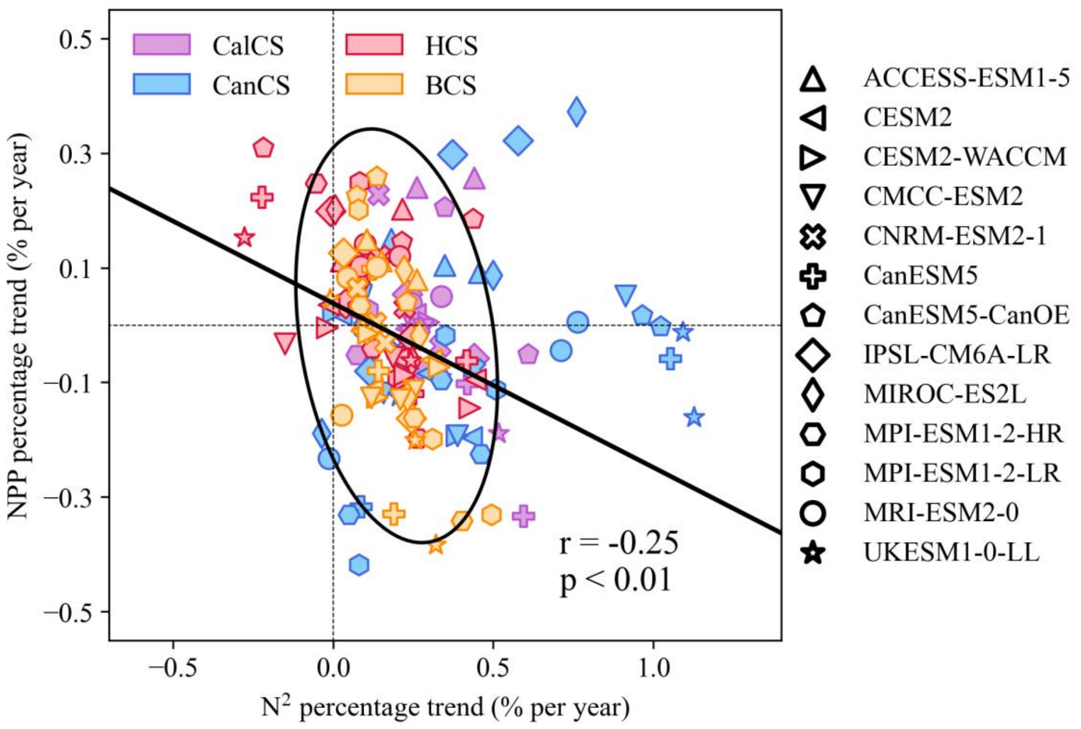

Supplementary Fig. 12. Relationship between Brunt-Väisäla frequency (N^2) and net primary production (NPP) within eastern boundary upwelling system (EBUS). Same as **Fig. 4b**, but showing the relationship between upper 150 m averaged Brunt-Väisäla frequency and NPP in CalCS (blue), Canary (CanCS, red), Humboldt (HCS, yellow), and Benguela (BCS, purple) Current Systems derived from Earth System Models.

Supplementary Table 1. List of climate simulations used in this study.

Models	Oceanic resolution (km)	Vertical velocity	Primary productivity
ACCESS-CM2	100	√	
ACCESS-ESM1-5	100	√	√
CAMS-CSM1-0	100	√	
CAS-ESM2-0	100	√	
CESM2	100	√	√
CESM2-WACCM	100	√	√
CIESM	50	√	
CMCC-ESM2	100	√	√
CNRM-CM6-1	100	√	
CNRM-ESM2-1	100	√	√
CanESM5	100	√	√
CanESM5-CanOE	100	√	√
E3SM-1-0	50	√	
E3SM-1-1	50	√	
E3SM-1-1-ECA	50	√	
EC-Earth3-Veg-LR	100	√	
FGOALS-f3-L	100	√	
FGOALS-g3	100	√	
FIO-ESM-2-0	100	√	
GISS-E2-1-G	100	√	
GISS-E2-2-G	100	√	
HadGEM3-GC31-LL	100	√	
IPSL-CM6A-LR	100	√	√
MIROC-ES2H	100	√	
MIROC-ES2L	100	√	√
MIROC6	100	√	
MPI-ESM1-2-HR	50	√	√
MPI-ESM1-2-LR	250	√	√
MRI-ESM2-0	100	√	√
NESM3	100	√	
NorESM2-LM	100	√	√
UKESM1-0-LL	100	√	√

Supplementary References

1. Rykaczewski, R. R. *et al.* Poleward displacement of coastal upwelling-favorable winds in the ocean's eastern boundary currents through the 21st century. *Geophys. Res. Lett.* **42**, 6424–6431 (2015).
